# Validation of Endogenous Control Genes by Real-Time Quantitative Reverse Transcriptase Polymerase Chain Reaction for Acute Leukemia Gene Expression Studies

**DOI:** 10.3390/genes15020151

**Published:** 2024-01-24

**Authors:** Flávia Melo Cunha de Pinho Pessoa, Vitória Beatriz de Jesus Viana, Marcelo Braga de Oliveira, Beatriz Maria Dias Nogueira, Rodrigo Monteiro Ribeiro, Deivide de Sousa Oliveira, Germison Silva Lopes, Ricardo Parente Garcia Vieira, Manoel Odorico de Moraes Filho, Maria Elisabete Amaral de Moraes, André Salim Khayat, Fabiano Cordeiro Moreira, Caroline Aquino Moreira-Nunes

**Affiliations:** 1 Department of Medicine, Pharmacogenetics Laboratory, Drug Research and Development Center (NPDM), Federal University of Ceará, Fortaleza 60430-275, CE, Brazil; flaviamelop@outlook.com (F.M.C.d.P.P.); bmdnogueira@gmail.com (B.M.D.N.); deividearmorial@gmail.com (D.d.S.O.); odorico@ufc.br (M.O.d.M.F.);; 2 Department of Biological Sciences, Oncology Research Center, Federal University of Pará, Belém 66073-005, PA, Brazil; vitoriavianabiomed@gmail.com (V.B.d.J.V.); oliveira.mb23@gmail.com (M.B.d.O.); fabiano.ufpa@gmail.com (F.C.M.); 3 Department of Hematology, Fortaleza General Hospital (HGF), Fortaleza 60150-160, CE, Brazil; 4 Department of Hematology, César Cals General Hospital, Fortaleza 60015-152, CE, Brazil; germison@gmail.com; 5 Department of Hematology, São Vicente de Paulo Maternity Hospital, Barbalha 63180-000, CE, Brazil; ricardopgv@gmail.com; 6 Central Unity, Molecular Biology Laboratory, Clementino Fraga Group, Fortaleza 60115-170, CE, Brazil

**Keywords:** leukemia, reference genes, RT-qPCR, gene expression

## Abstract

Reference genes are used as internal reaction controls for gene expression analysis, and for this reason, they are considered reliable and must meet several important criteria. In view of the absence of studies regarding the best reference gene for the analysis of acute leukemia patients, a panel of genes commonly used as endogenous controls was selected from the literature for stability analysis: Glyceraldehyde-3-phosphate dehydrogenase (*GAPDH*), Abelson murine leukemia viral oncogene human homolog 1 (*ABL*), Hypoxanthine phosphoribosyl-transferase 1 (*HPRT1*), Ribosomal protein lateral stalk subunit P0 (*RPLP0*), β-actin (*ACTB*) and TATA box binding protein (*TBP*). The stability of candidate reference genes was analyzed according to three statistical methods of assessment, namely, NormFinder, GeNorm and R software (version 4.0.3). From this study’s analysis, it was possible to identify that the endogenous set composed of *ACTB*, *ABL*, *TBP* and *RPLP0* demonstrated good performances and stable expressions between the analyzed groups. In addition to that, the *GAPDH* and *HPRT* genes could not be classified as good reference genes, considering that they presented a high standard deviation and great variability between groups, indicating low stability. Given these findings, this study suggests the main endogenous gene set for use as a control/reference for the gene expression in peripheral blood and bone marrow samples from patients with acute leukemias is composed of the *ACTB*, *ABL*, *TBP* and *RPLP0* genes. Researchers may choose two to three of these housekeeping genes to perform data normalization.

## 1. Introduction

The standard polymerase chain reaction (PCR) was developed in the 1980s by Kary Mullis and resembles an in vitro elementary form of DNA replication, mimicking the physiological process that occurs in living organisms. Up to the present time, many variations of this technique have been developed, such as Nested-PCR, reverse transcriptase PCR (RT-PCR), real-time quantitative reverse transcriptase PCR (RT-qPCR) and others [1,2,3].

Real-time quantitative reverse transcriptase PCR (RT-qPCR) technology has revolutionized the detection landscape in every area of molecular biology. This technique is based on the conversion of an RNA template to complementary DNA (cDNA), followed by fluorescent reporter dye detection to measure the amplification at each PCR cycle with higher sensitivity and specificity [1,3,4,5,6,7,8].

The RT-qPCR technique is considered as the most reliable and most accurate method of diagnosis [9,10,11,12,13,14,15,16,17]. However, numerous failures can occur during the RT-qPCR process, which can lead to the misinterpretation of results and incorrect conclusions. That is why the application of an appropriate normalization method is an absolute necessity to achieve reliable results [18,19,20,21]. There are many ways to normalize RT-qPCR data, such as normalization with genomic DNA, total RNA, artificial molecule (spike) and reference genes [4,13,20,22,23,24].

The use of reference genes is considered one of the most effective methods for the normalization step of the RT-qPCR technique [13]. Reference genes may be used as an internal reaction control for gene expression analysis, and for this reason, they are considered reliable and must meet several important criteria. A good reference gene is unaffected by experimental factors and shows minimal variability between tissues and physiological states of the organism. Therefore, it is beneficial to choose a reference gene that shows a similar threshold cycle in studies within different genes of interest [17,18,25,26,27,28,29,30,31,32,33].

Basic metabolism genes often present the perfect fulfillment of these conditions since they are expressed at a stable and constant level and are involved in essential cell processes. Because of that, they can be called housekeeping genes (HKGs). In the past, the validation process was often avoided and HKGs were used due to a common belief that they are characterized by constant expression levels regardless of their conditions and origins. As awareness of the complex expression regulation networks in the cell function grew, this statement began to be undermined and experimental confirmation of the stability of candidate genes is now a standard requirement [14,15,34,35,36,37,38,39,40,41].

A relevant number of traits can have different impacts on gene expression, such as tissue type, developmental stage, related species, abiotic stress, diseases, infections, alternative splicing, and tumors. Therefore, it is observed that the need for reference gene validation has been underlined several times in different articles involving different types of diseases, especially in the oncological area, where types of cancer have different physiologies and the involvement of several genetic and external factors. Since cancer encompasses so many variables, molecular studies are required to determine comparative genes for expression analyses, which is imperative for adequate normalization, whose task is to compensate for PCR variations resulting from basic difficulties inherent to the method. Therefore, the ideal is to find genes with undoubtedly stable expression for each disease/condition model [42,43,44,45,46,47,48,49,50].

Several studies have already been carried out aiming to validate the reference genes for different types of cancer, allowing the normalization of gene expression analyses in future studies. McNeil et al. [47] validated the *MRPL19* and *PPIA* genes as endogenous controls for analysis of breast cancer patients. For the analysis of endometrial cancer, the most stable reference genes were *RPL30*, *MT-ATP6* and *ACTB* according to research conducted by Ayakannu et al. [51].

There are still no concrete published studies on the validation of reference genes using the RT-qPCR technique for gene expression analysis involving acute leukemia patients. Our group chose to perform validation on both peripheral blood and bone marrow samples, as these are the two sample types used in our laboratory for the detection of genetic alterations in acute leukemia patients through the RT-qPCR technique. This validation’s importance is due to the fact that this methodology is widely used at these patients’ time of diagnosis but also for the detection of minimal residual disease (MRD), with the ability to identify if they are having a good treatment response and/or if they are disease free at the end of the chemotherapy protocol. Therefore, it is extremely necessary to perform a reference gene normalization in this technique, so it is as accurate as possible.

Considering the importance of using the reference gene as a control for gene expression analysis and the lack of studies in the literature that validate the best reference genes to be used for the analysis of patients with acute leukemias, this work aimed to perform this validation and normalize expression assays with peripheral blood and bone marrow samples from these patients.

## 2. Materials and Methods

### 2.1. Biological Samples

All adult patients with AML (24) and ALL (25) participating in this study were treated at the Fortaleza General Hospital (Fortaleza, CE, Brazil) and sought care due to suspicion of the disease, i.e., samples from all patients were collected during the diagnostic phase. Samples were collected prospectively from July 2021 to July 2022. Control group samples (15) were also collected during the same period. Pediatric ALL samples (25) were collected from 2012 to 2023 at Octávio Lobo Children’s Hospital (Belém, PA, Brazil), also in the diagnostic phase (Table 1). The diagnosis of these patients was made in the participating hospitals and local blood centers through tests such as myelogram, immunophenotyping and karyotyping.

Patients’ samples were collected in ethylenediaminetetraacetic acid (EDTA) collection tubes at the time of diagnosis and were packed in a thermal case at 2–4 °C for transport to the laboratory for later processing. After collection, the samples were immediately processed, going through the buffy coat separation for RNA extraction and conversion into cDNA. The cDNA samples were stored until the end of the collections, in July 2022, in a freezer (−2 °C to −8 °C) for RT-qPCR assays.

This study was approved by both the Ethics Committee of the Ophir Loyola Hospital (approval number: 2,798,615) and the Ethics Committee of the General Hospital of Fortaleza (approval number 4,798,575). Informed written consent was obtained from the patients or the patients’ legal guardians, and all methods were carried out in accordance with Helsinki guidelines and regulations.

### 2.2. RNA Extraction and Reverse Transcription of RNA to cDNA

RNA from samples was extracted with TRIzol Reagent^®^ (Invitrogen, Waltham, MA, USA) according to the manufacturer’s instructions. From 20 μL of RNA, the cDNA was synthesized using a High-Capacity cDNA Reverse Transcriptase kit (Life Technologies, Carlsbad, CA, USA) to convert the extracted and purified RNA to cDNA. The conversion step was performed on a Veriti^®^ Thermal Cycler (Applied Biosystems, Foster City, CA, USA). After this step, the samples were stored in a freezer at −20 °C until use for analysis.

### 2.3. Identification of Gene Expression by Quantitative Real-Time Polymerase Chain Reaction (qPCR)

Quantitative real-time polymerase chain reaction was performed to evaluate the endogenous expressions that are commonly used in studies of general gene expression. Information about the genes and probes chosen for the study is shown in Table 2. Regarding the protocol, for each sample, the following were used: 1 μL of cDNA, 0.5 μL of each primer/probe, 5 μL of TaqMan^®^ Gene Expression Master Mix (Life Technologies, Carlsbad, CA, USA) and 3.5 μL of ultra-pure water. RT-qPCR was performed for the following genes shown in Table 2, and each sample was analyzed in triplicate for experimental and technique validation, according to the international standards for evaluation of gene expression by real-time PCR [46,52].

The gene-expression levels were based on absolute and relative analyses and calculated using the 2^−∆∆CQ^ (delta delta cycle quantification) method, using the healthy samples as the calibrator/control [46,52]. Fold change data are represented as mean ± standard deviation of three independent experiments.

### 2.4. Data Analysis Based on the Gene Expression Omnibus (GEO) Database

We used the Gene Expression Omnibus (GEO) (https://www.ncbi.nlm.nih.gov/gds (accessed on 12 July 2023)), a public repository of high-throughput gene expression data, as a reference model to profile *ACTB*, *ABL*, *GAPDH*, *HPRT*, *TBP* and *RPLP0* expression in acute leukemias. The spreadsheet created with all the Ct (cycle threshold) data of all endogenous genes and all the analyzed samples is available as a Supplementary file. The file contains 4 spreadsheets: metadata template, matrix non-normalized template, matrix normalized template and fold change template. The metadata template spreadsheet contains the description of all samples used in the study, such as the numbering of each patient, leukemia type and sample type information. The matrix non-normalized template spreadsheet reports all the raw Ct averages of each analyzed sample, where the rows correspond to the reference genes used in the research and the columns correspond to the samples tested. The matrix normalized template spreadsheet contains target gene signals normalized to housekeeping genes, e.g., 2^−ΔCt^, where −ΔCt = −(Ct_Target − Ct_HKG), where the rows correspond to the reference genes used in the research and the columns correspond to the samples tested. And finally, the last spreadsheet, fold change template, reports the fold change data, e.g., SAMPLE_test_ target gene signal normalized to housekeeping gene (2^−ΔCttest^)/SAMPLE_control_ target gene signal normalized to HKG (2^−ΔCtcontrol^), where the rows correspond to the reference genes used in the research and the columns correspond to the samples tested.

### 2.5. Statistical Analysis

The stability of candidate reference genes was analyzed according to three statistical methods of assessment, namely, the Delta Ct method, the estimation of the intra- and intergroup variation (NormFinder) [53] and pairwise comparison (GeNorm) [43]. In addition, a deeper and more individual analysis was carried out using a Kruskal–Wallis (KKW) test in the R software [54], and comprehensive ranking orders of these candidate genes were available from the four methods.

GeNorm uses a pairwise comparison-based model to select, from a panel of candidate HKGs, the gene pair showing the least variation in expression ratio across the samples. It calculates a measure of gene stability (M) of each gene based on the average pairwise variation between all tested genes. Genes with the lowest M values are those demonstrating the most stable expression. This calculation is based on the principle that it stepwise excludes the gene with the highest M value. In addition, GeNorm involves a cut-off value of 0.15, below which the inclusion of an additional reference gene is not required. This cut-off value of 0.15 is suggested by Vandesompele et al. (2002) when multiple control genes are used as a normalization factor [43].

NormFinder uses a model-based approach to estimate not only the overall expression variation of the candidate normalization genes but also the variation between the sample subgroups of the sample set. The candidates with the lowest intergroup and intragroup variations give the lowest S stability value and are therefore ranked higher as more stable [53].

The R software (https://www.r-project.org, accessed on 14 September 2023) allows a deep analysis. To evaluate the potential endogenous genes, three analyses were performed: (i) using all samples, (ii) using only peripheral blood (PB) samples and (iii) using only bone marrow (BM) samples. 

The Kruskal–Wallis (KKW) test was performed for each gene to identify significant differences among the four groups (control, AML, ALL and ALL_Ped). Additionally, the sum of the square differences between each condition mean (Ct) and standard deviation and the gene mean (Ct) and standard deviation were calculated. These values indicate the mean and standard deviation variation among different groups. Greater values indicate more variance among groups [55,56].

**Figure 1:**
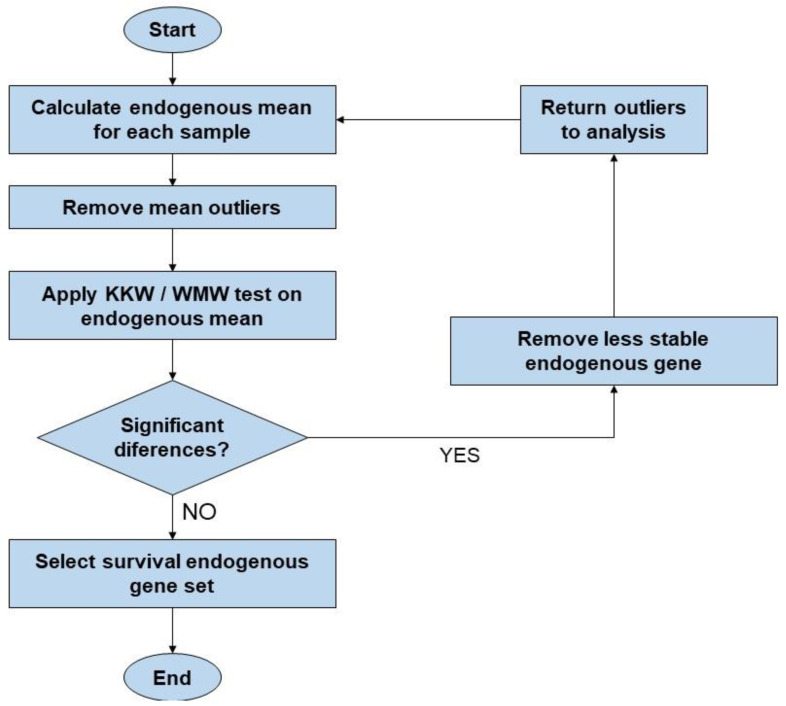
Flowchart of the steps used to select the best endogenous gene set. This figure illustrates the methodology utilized during this study’s statistical analysis in the R software. KKW: Kruskal–Wallis; WMW: Wilcoxon–Mann–Whitney.

To select the best endogenous gene set, we utilized an interactive methodology involving multiple steps: (i) we calculated the endogenous mean for each sample; (ii) we excluded outlier samples (|MeanCt| > 2); (iii) the Kruskal–Wallis test was applied to assess variations in the endogenous means across different conditions; (iv) in cases where significant differences were identified among conditions or groups, the least stable endogenous gene was systematically removed from the analysis and the excluded samples removed in step (ii) were then reintegrated, and the process recommenced from step (i); (v) if there are no significant differences among groups, the survival endogenous gene set is selected (Figure 1).

To identify the less stable endogenous gene, the following criteria were applied: (i) gene significant differences among conditions; (ii) standard deviation values; (iii) sum of the square differences of the mean and standard deviation between conditions/groups and the gene; (iv) NormFinder and GeNorm stability values.

To evaluate the variation of potential endogenous genes in ALL and AML groups in different origin samples (PB and BM), a two-way ANOVA ranking was used. The statistical significance threshold was set at *p*-value < 0.05 for all analyses [57,58,59]. 

## 3. Results

In order to identify the best reference genes for gene expression studies in peripheral blood and/or bone marrow samples of acute leukemia patients, a qPCR assay based on TaqMan detection for the expression analysis of the six selected genes (*ACTB*, *ABL*, *GAPDH*, *HPRT*, *TBP* and *RPLP0*) was used.

The gene ranking according to GeNorm indicates that *TBP* is the gene with the most stable expression for adult ALL and AML patients, with stability values of 0.098 and 0.073, respectively, and *HPRT* is the gene with the most stable expression for both pediatric ALL patients and normal samples, with a stability value of 0.059 and 0.040, respectively (Table 3). 

NormFinder identified a different result from GeNorm, probably because it was possible to identify the different sample groups during the analysis. The *ACTB* gene was the most stable gene followed by the RPLP0 gene, which had stability values of 0.47 and 0.52, respectively. The least stable reference gene candidate was *GAPDH* with a stability value of 1.78 (Table 4).

The R software allowed a deep analysis, as this tool is capable of performing intergroup and intragroup analyses, ensuring a more adequate evaluation of the endogenous genes. Table 5 summarizes the results obtained through the analyses performed in the R software, including Kruskal–Wallis results, gene standard deviation, sum of mean square difference and sum of standard deviation square difference. Among all the results, we highlight that it was possible to observe that the *ACTB* gene presented a very good performance with low mean and standard deviation values (1.52 and 1.69, respectively), as well as little difference in the standard deviation between all groups (Table 5).

The analysis made in this study showed that the *GAPDH* and *HPRT* genes could not be classified as good reference genes, considering that they presented a high standard deviation and great variability between groups, indicating low stability. From these results, it can be considered that *GAPDH* and *HPRT* do not behave as good endogenous genes for the expression analysis of acute leukemia samples; therefore, they should not be used.

**Figure 2:**
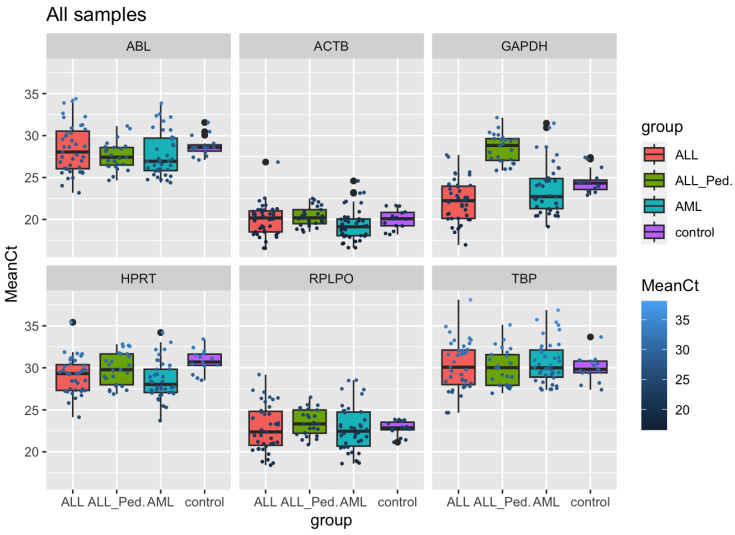
Box-plot graph indicating the range of Ct values of the reference genes by group. ALL: adult acute lymphoblastic leukemia; ALL_Ped: pediatric acute lymphoblastic leukemia; AML: acute myeloid leukemia. This figure, obtained through R software analysis, illustrates the mean Ct of each endogenous candidate in the different groups analyzed, allowing the visualization of which endogenous genes showed greater expression instability and which were more stable. The smaller the mean Ct variation and the smaller the variation between the groups, the more stable the endogenous gene evaluated.

**Figure 3:**
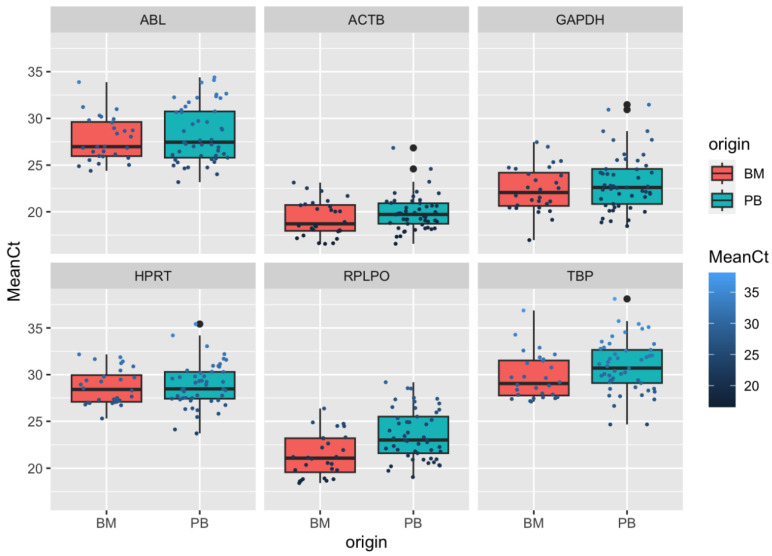
Box-plot graph indicating the range of Ct values of reference genes by sample origin. BM: bone marrow; PB: peripheral blood. This figure, obtained through R software analysis, illustrates the mean Ct of each endogenous candidate according to the type of sample analyzed (bone marrow and peripheral blood). Only the AML and adult ALL groups participated in this analysis since they were the only two groups that had both sample types.

In addition, it was possible to identify that the endogenous gene set composed of *ACTB*, *ABL*, *TBP* and *RPLP0* demonstrated good performances and stable expressions between the analyzed groups. The expression levels for many of these genes fluctuate dramatically both within and across datasets (Figure 2). The origin of the sample (peripheral blood or bone marrow) may also have an influence on the reference gene expression (Figure 3).

As most endogenous gene validation studies report, we have observed, in our study, an expression variation of endogenous candidates in the different types of samples tested (bone marrow and peripheral blood). However, in general, the expression variation observed was not as high. In Figure 3, it is possible to identify that the endogenous genes *ACTB* and *ABL* are the two most suitable for studies that rely on both types of samples since there is a lower variation in expression between bone marrow and peripheral blood. If the study relies only on peripheral blood samples, researchers can choose two or three endogenous genes from the *ACTB*, *ABL*, *TBP* and *RPLP0* gene set.

## 4. Discussion

The normalization of gene expression in a group of samples is necessary to validate the stability of the expression of a reference gene under experimental conditions, such as sample type and disease type, for example, before its use in studies. In the literature, it is possible to observe that most works use the standardization programs GeNorm and NormFinder to search for the most appropriate reference genes for their studies. However, although these tools are capable of analyzing endogenous genes in different groups to try to identify specific behaviors for each of them, they do not conduct it in a completely satisfactory way [51,60,61,62,63].

In studies of normalization and validation of endogenous genes to disease models, the statistical test must be performed between endogenous Ct-case and endogenous Ct-control. If there is a large difference between both Cts, the use of the endogenous gene in question is not feasible, since it is being influenced by the conditions. Furthermore, it was observed during our statistical analysis that it is necessary to remove assays whose endogenous gene presents outlier behavior (endogenous Ct > 2 × SD of the average of endogenous Cts). It is of great importance to verify whether there is a correlation between endogenous Ct and target Ct, considering that it may characterize experimental bias.

Since this work had four distinct groups, pediatric ALL, adult ALL, adult AML, and control, checking the data through these tools, it was possible to perceive that when they were analyzed all together, there was a loss of intragroup variability; on the other hand, when the analyzes were performed separately by group, there was a loss of intergroup variability. Thus, it was not possible to actually elect the most appropriate endogenous gene using these tools for the proposed study. For this reason, additional analyses were performed using the R software [43,53,54,64].

Currently, the most frequently used reference genes for general expression studies are B-actin (*ACTB*), glyceraldehyde-3- phosphate dehydrogenase (*GAPDH*) and hypoxanthine-guanine phosphoribosyl transferase 1 (*HPRT1*) [13,65,66,67,68,69].

The *GAPDH* gene is involved in many cell processes such as membrane transport and membrane fusion, microtubule assembly, nuclear RNA export, protein phosphotransferase/kinase reactions, DNA replication and DNA repair. With this in mind, *GAPDH* expression would be expected to vary as it has a diverse range of functions unrelated to its glycolytic activity [70].

This study’s analysis determined that the *GAPDH* gene presented the most unstable behavior between the analyzed endogenous genes. Our findings agree with several other studies that have scrutinized the stability of the commonly known reference gene *GAPDH* and have demonstrated that it should be used with caution as its expression varied considerably, and it was consequently unsuitable as a reference gene in some cases [12,70,71,72,73]. However, some studies have shown different results regarding the expression stability of *GAPDH*, as it was identified as one of the best housekeeping genes in the analysis of a great variety of tissue type [74,75,76].

The *HPRT* gene is also widely used as an endogenous control in many studies of gene expression in different types of cancer. This gene is found in all cells as a soluble cytoplasmic enzyme. Although *HPRT* is found in all types of somatic cells, significantly higher levels are found in the central nervous system [77,78]. Many studies have shown that the *HPRT* gene presents the behavior of a good reference gene, both used alone and associated with other genes such as *TBP* and *GAPDH*, among others [11,71,79,80,81,82].

A study by Jacques B. de Kok and colleagues chose *HPRT* as the reference gene with the highest accuracy when used as a single normalization gene in several types of solid tumors, such as colorectal, breast, prostate, skin, and bladder tissues, with tumors ranging from noninvasive to metastatic carcinomas. In this study, the authors analyzed 13 housekeeping genes (*LRP*, *ACTB*, *CYC*, *GAPDH*, *PGK*, *B2M*, *BGUS*, *HPRT*, *TBP*, *TfR*, *PBGD*, *ATP6* and *rRNA*) in order to elect which one was most stable [83].

However, our study also identified that the *HPRT* gene presents low stability in samples of patients with acute leukemias and is not indicated as a suitable endogenous gene. Some other studies have also reported that the *HPRT* gene exhibits high expression variability and have classified it as an inadequate reference gene, corroborating the data found in this work [84,85].

By using the previously described method, the endogenous genes *GAPDH* and *HPRT* were removed from the analysis due to poor performance. Both genes presented high standard deviation and high variability between the analyzed groups, characterizing bad behavior for a reference gene. Therefore, our study proposes the set of endogenous genes *ACTB*, *ABL*, *TBP* and *RPLP0* as the most appropriate for the analysis of expression assays of acute leukemia samples.

The *ACTB* gene is an abundant and highly conserved cytoskeleton structural protein that is widely distributed in all eukaryotic cells and that plays critical roles in multiple cell processes. It is usually regarded as a constitutive housekeeping gene, assuming that its expression is normally unaffected by most experimental or physiological conditions. Therefore, *ACTB* has been widely used as a reference gene for expression analysis in many types of tissues [10,35,86,87].

In this study, the *ACTB* gene was reported as one of the most stable endogenous genes analyzed, presenting low mean and standard deviation values intragroup and between all four groups. This gene has also been reported as a good reference gene in other studies of different types of cancers, but especially in breast cancer expression analysis [47,88,89,90].

However, the *ACTB* gene was found to be differentially expressed in many different types of cancer such as liver, melanoma, renal, colorectal, gastric, pancreatic, esophageal, lung, breast, prostate, ovarian cancers, leukemia, and lymphoma under certain conditions. This suggests that it might be an unsuitable endogenous gene for expression analysis [9,87,91,92]. Some studies reported *ACTB* as an unsuitable reference gene [67,71,73,93,94].

In a recent study, Gupta and colleagues performed an identification and validation of the optimal reference genes for standardizing the gene expression profiling diagnostic panel of Ph-like B-lineage acute lymphoblastic leukemia. They reported that *EEF2*, *GAPDH* and *PGK1* are optimal and stable endogenous genes for specific gene quantification in Ph-like ALL cases as compared to *ABL1*, *ACTB*, *B2M*, *RNA18S*, *GUSB* and *TBP* [95].

Two other genes that showed stable gene expression were two protein-coding genes, *ABL* and *TBP*, respectively, according to mean values and standard deviation. The *ABL* gene is an oncogene likely associated with many roles of cell cycle regulation, stress responses, integrin signaling and neural development [96,97,98]. The TATA-binding protein (*TBP*), in turn, has been considered a universal transcription factor that is required for initiation by all three nuclear RNA polymerases, and it is also a component of the DNA-binding protein complex of transcription factor II D (TFIID). This gene is associated with a variety of factors that play important roles in the regulation of gene expression [10,99].

The *ABL* gene was constantly expressed in the peripheral blood of healthy individuals at levels comparable to other analyzed reference genes in different studies, including studies with chronic myelogenous leukemia (CML) expression analysis [100,101,102]. Furthermore, a study published by Weisser et al. in 2004 reported that *ABL* was a suitable endogenous gene for monitoring minimal residual disease in acute myeloid leukemia patients [103].

Altogether, the data from many different studies show the relevance of *TBP* gene expression stability, indicating that it is a suitable reference gene to be used as a control in studies of various kinds of diseases, including some types of cancer such as bladder cancer and glioblastoma. However, the majority of these studies also showed that the use of *TBP* associated with other reference genes presented an even better performance [92,93,104,105,106,107,108].

*RPLP0* is a ribosomal protein that is responsible for recruiting both translation factors and other ribosomal proteins to the ribosomal complexes, facilitating protein synthesis. Usually, it is strongly expressed in normal lymph nodes, skin, spleen and fetal brain tissue, expressed at lower levels in normal lung, bladder and placenta and not expressed in normal colon, kidney and bone marrow [109,110,111].

What was observed in this study in relation to the *RPLP0* gene is what is usually demonstrated in the other endogenous gene validation studies for gene expression techniques. The *RPLP0* gene presents a relatively good expression stability in several studies, but it is not the most suitable reference gene [44,112,113,114,115].

Table 6 summarizes all the endogenous gene normalization and validation studies that were mentioned in the discussion of this work, allowing for better data visualization, such as model (tissue type), number of patients, analyzed samples, endogenous genes tested, endogenous gene with best behavior and year of each study. Through this table, it is possible to observe that most reference genes’ stability varies among different disease models, demonstrating that performing this kind of validation study before any gene expression analysis is very important.

## 5. Conclusions

Given these findings, this study suggests the main endogenous gene set for use as a control/reference for the analysis of gene expression in peripheral blood and bone marrow samples from patients with acute leukemias is composed of the *ACTB*, *ABL*, *TBP* and *RPLP0* genes. Researchers may choose two to three of these endogenous controls to perform data normalization.

In addition, the statistical analysis in this type of study is indispensable. It is important to verify the variation of endogenous gene expression between groups. Once the calculation of delta Ct is made, the variability of endogenous gene expression is transferred to the target. Then, if there is a high endogenous gene variability between groups, potential differences found on the target may not really indicate target variation but actually the influence of the endogenous variation.

Therefore, it is extremely necessary to perform the reference gene validation for any gene expression study, considering that the endogenous gene used influences the reliability and accuracy of these studies.

## Figures and Tables

**Table 1:** Samples examined in this study and patients’ characteristics.

	Number of Patients	Bone Marrow	Peripheral Blood	Mean Age (Range)	Median Age	Gender
AML	24	14	24	51.1 (19–96)	47.5	F: 9 M: 15
ALL	25	14	25	42.5 (19–96)	36	F: 8 M: 17
ALL_Ped	25	-	25	8 (0–18) *	7 *	F: 8 M: 14 NA: 3
Control	15	-	15	33.7 (20–47)	33	F: 10 M: 5
Total	89	28	89			

AML: acute myeloid leukemia; ALL: adult acute lymphoblastic leukemia; ALL_Ped: pediatric acute lymphoblastic leukemia; F: female; M: male; NA: not available. * These numbers were calculated with only 22 patients’ age information since the remaining 3 were not available.

**Table 2:** Selected endogenous genes for quantitative real-time PCR evaluation.

Gene Symbol	Gene Name	Chromosome Location	Function	Amplicon Size	Assay Number
*ABL1*	Abelson murine leukemia viral oncogene human homolog 1	Chr.9: 130713881–130887675	Protein tyrosine kinase involved in a variety of cellular processes	60	Hs01104728_m1
*ACTB*	β-actin	Chr.7: 5527148–5530601	Cytoskeletal structural protein	63	Hs01060665_g1
*GAPDH*	Glyceraldehyde-3-phosphate dehydrogenase	Chr.12: 6534405–6538375	Oxidoreductase in glycolysis and gluconeogenesis	157	Hs02786624_g1
*HPRT1*	Hypoxanthine phosphoribosyl-transferase 1	Chr.X: 134460165–134500668	Purine synthesis in salvage pathway	82	Hs2800695_m1
*RPLP0*	Ribosomal protein lateral stalk subunit P0	Chr.12: 120196700–120201211	Ribosomal protein translation	76	Hs00420895_gH
*TBP*	TATA box binding protein	Chr.6: 170554333–170572870	Regulation of transcription DNA and component of the DNA-binding protein complex TFIID	91	Hs00427620_m1

**Table 3:** GeNorm endogenous gene stability value (M).

	*ABL*	*ACTB*	*GAPDH*	*HPRT*	*TBP*	*RPLP0*
ALL	0.113	0.104	0.128	0.100	0.098	0.121
AML	0.087	0.083	0.090	0.098	0.073	0.091
ALL_Ped	0.059	0.066	0.082	0.059	0.060	0.071
Control	0.045	0.052	0.067	0.040	0.044	0.044

GeNorm program did not allow the identification of the different groups in its analyses; therefore, each group was analyzed separately.

**Table 4:** NormFinder endogenous gene stability value ranking.

	Gene	Stability Value
Overall *		
1	*ACTB*	0.47
2	*RPLP0*	0.52
3	*HPRT*	0.72
4	*ABL*	0.86
5	*TBP*	0.91
6	*GAPDH*	1.78

* The NormFinder normalizer program allowed a single analysis of all study samples subdivided by groups (AML, ALL, ALL_Ped and control), identifying the reference genes with the best overall performance.

**Table 5:** Software R endogenous candidates’ analysis.

	*ABL*	*GAPDH*	*ACTB*	*HPRT*	*TBP*	*RPLP0*
Kruskal–Wallis *p*-value	0.176	0.000	0.046	0.000	0.914	0.368
Gene Standard Deviation	2.421	3.352	1.698	2.150	2.472	2.362
Sum of Mean Square Difference	0.893	22.500	0.487	3.256	0.286	0.385
Sum of Standard Deviation Square Difference	2.448	7.396	0.626	0.715	1.223	3.170

**Table 6:** Analysis of the previous studies of normalization and validation of endogenous genes compared to this current study.

Model (Tissue Type)	Number of Patients	Analyzed Sample	Endogenous Genes Tested	Endogenous Genes with Best Behavior	Year	Reference
Acute leukemias	89	Peripheral blood and bone marrow	*ACTB*, *ABL*, *GAPDH*, *HPRT1*, *TBP* and *RPLP0*	*ACTB*, *ABL*, *TBP* and *RPLP0*	2024	Present study
Acute myeloid Leukemia	29	Peripheral blood	*ABL1*, *G6PDH*, *B2M* and *PBGD*	*G6PDH* and *ABL*	2004	[103]
ALL, Ph-like B-lineage	23	Peripheral blood	*ABL1*, *GUSB*, *EEF2*, *18S*, *ACTB*, *GAPDH*, *TBP*, *PGK1*, *B2M*, *JCHAIN*, *SPATS2L*, *CA6*, *NRXN3*, *MUC4*, *CRLF2*, *ADGRF1* and *BMPR1B*	*EEF2*, *GAPDH* and *PGK1*	2023	[95]
B-cell chronic lymphocytic leukemia	30	Peripheral blood	*ACTB*, *B2M*, *GAPDH*, *GUSB*, *HMBS*, *HPRT1*, *MRPL19*, *TBP* and *UBC*	*B2M*, *HPRT1* and *GUSB*	2010	[71]
Bladder and colon cancer	58	Tumor biopsies	*FLOT2*, *ATP5B*, *HSPCB*, *S100A6*, *TEGT*, *CFL1*, *FLJ20030*, *TPT1*, *UBB*, *TBC*, *RPS23*, *GAPD*, *ACTB*, *CLTC*, *NACA*, *SU11* and *TUBA6*	*UBC*, *GAPD* and *TPT1* for colon and *HSPCB*, *TEGT* and *ATP5B* for bladder	2004	[53]
Bladder cancer	14	Tissue biopsies	*ACTB*, *ALAS1*, *G6PD*, *GAPD*, *HMBS*, *HPRT1*, *K-α-1*, *SDHA* and *TBP*	*SDH* and *TBP*	2006	[106]
Breast cancer	87	Tumor biopsies and cell lines	*SF1*, *TARDBP*, *THRAP3*, *QRICH1*, *TRA2B*, *SRSF3*, *YY1*, *DNAJC8*, *RNF10* and *RHOA*	*SF1*, *TRA2B*, *THRAP3*, *RHOA* and *QRICH1*	2021	[60]
Breast cancer	-	Cell line (MCF-10A)	*18S*, *28S*, *ACTB*, *PPIA*, *GAP* and *RPL32*	*18S* and *ACTB*	2005	[88]
Breast cancer	40	Tumor biopsies	*GAPDH*, *TFRC*, *RPLP0*, *GUSB*, *HPRT1*, *UPA* and *ACTB*	*ACTB* and *TFRC*	2011	[89]
Breast cancer	23	Tumor biopsies	*ACTB*, *GAPD*, *TBP*, *SDHA*, *HPRT*, *HMBS*, *B2M*, *PPIA*, *GUSB*, *YWHAZ*, *PGK1*, *RPL41*, *PUM1*, *RPLP0*, *MRPL19*, *TTC22*, *IL22RA1* and *ZNF224*	*ACTB* and *SDHA*	2009	[90]
Breast, gastric, esophageal, colon, rectum, and lung carcinomas	327	Tissue biopsies	*ACTB*, *GAPDH*, *GUSB*, *RPLPO* and *TFRC*	The optimal reference genes were tissue-specific	2014	[115]
Brown adipose tissue	-	Tissue biopsies	*18S*, *B2M*, *GAPDH*, *LRP10*, *PPIA*, *RPLP0*, *UBC* and *YWHAZ*	*PSMB2*, *GNB2* and *GNB1*	2015	[45]
Cancer stem cells	-	Cell lines (RD, MG63, HOS, Saos-2, A673, MDA-MB-231 and ACHN)	*18S*, *ACTB*, *B2M*, *G6PD*, *GAPDH*, *GUSB*, *HMBS*, *HPRT1*, *PGK1*, *PPIA*, *RPL13a*, *SDHA*, *TBP*, *TUBB* and *YWHAZ*	*GAPDH*, *TBP* and *PPIA*	2016	[93]
Cervical cancer	-	Cell lines (SiHa, HeLa and ME180)	*ACTB*, *B2M*, *GAPDH*, *HPRT1* and *TBP*	*B2M*, *GAPDH*, *HPRT1* and *TBP*	2018	[62]
Colon	-	Rat tissue biopsies	*GAPD*, *ACTB*, Cyclophilin A, *HPRT*, *AcRP0*, *L32*, *18S* and *28S*	*AcRP0*	2004	[82]
Colon, breast, prostate, skin, and bladder	16	Tissue biopsies	*LRP*, *BACT*, *CYC*, *GAPDH*, *PGK*, *B2M*, *BGUS*, *HPRT*, *TBO*, *TfR*, *PBGD* and *ATP6*	*HPRT*	2005	[83]
Colon, liver, pancreas, rectum, lung, cervix, ovary, prostate, umbilical, breast, spleen, etc.	72	Tissue biopsies	*GAPDH*	*GAPDH* varies a lot between tissues	2005	[76]
Colorectal cancer	64	Peripheral blood	*ACTB*, *B2M*, *GAPDH*, *HPRT1*, *SDHA TBP*, *IL-1B* and *CCL4*	*HPRT1*, *SDHA* and *TBP*	2020	[73]
Endometrial cancer (type 1 or type 2)	15	Endometrial biopsies	*RPL30*, *MT-ATP6*, *18S*, *ACTB*, *TBP*, *RPLP0*, *PES1*, *POLR2A*, *TFRC*, *HPRT1*, *ABL1*, *GADD45A*, *HMBS*, *CDKN1A*, *RPL37A*, *UBC*, *GAPDH*, *CDKN1B*, *CASC3*, *POP4*, *PGK1*, *GUSB*, *YWHAZ*, *PPIA*, *RPS17*, *MRPL19*, *B2M*, *EIF2B1*, *ELF1*, *PSMC4*, *PUM1* and *IPO8*	*PSMC4*, *PUM1* and *IPO8* for type 1 and *UBC*, *MRPL19*, *PGK1* and *PPIA* for type 2	2020	[51]
Glioblastoma	30	Tumor biopsies	*ACTB*, *GAPDH*, *GUSB*, *HMBS*, *HPRT1*, *TBP*, *18S*, *TG1*, *TG2*, *TG3*, *TG4*, *TG5*, *TG6*, *TG7*, *TG8*, *GT9*, *TG10*, *TG11* and *TG12*	*TBP* and *HPRT1*	2009	[108]
Hepatocellular carcinoma	65	Tumor biopsies	*ACTB*, *GAPDH*, *B2M*, *HPRT1* and *TBP*	*TBP* and *HPRT*	2008	[80]
Hippocampal tissue	25	Tissue biopsies	*ACTB*, *GAPDH*, *HPRT*, *NSE*, *SDHA* and *SYP*	*HPRT*, *NSE*, *SDHA* and *SYP*	2012	[81]
Hypoxia and hyperglycemia model	-	Umbilical cords	*RPLP0*, *GAPDH*, *GUSB*, *TFRC* and *ACTB*	*TFRC* and *RPLP0*	2014	[114]
Lung cancer	-	Cell lines (A549, NCI-H446 and NCI-H460)	*18S*, *GAPDH*, *RPLP0*, *ACTB*, *PPIA*, *PGK1*, *B2M*, *RPL13A*, *HPRT1* and *TBP*	*ACTB*, *PPIA* and *PGK1*	2015	[113]
Lung, breast, colon, prostate, and pancreas	326	Tissue biopsies	*HPRT*	*HPRT* should no longer be used as an endogenous standard	2019	[84]
Lymphoid malignancies	92	Cell lines, tumor biopsies and peripheral blood	*18S*, *RPLP0*, *GAPD*, *PPIA*, *PRKG1*, *TBP*, *ACTB*, *B2M* and *GUSB*	*PRKG1* and *TBP*	2003	[104]
Melanoma	-	Cell lines (IC8 and T1C3)	*GAPDH*, *18S* and *ACTB*	*18S*	2001	[91]
Myoblasts	15	Tissue biopsies	*ACTA1*, *MYOG*, *MYH3*, *ACTB*, *B2M*, *GAPDH*, *PPIA*, *RPLP0* and *TBP*	*RPLP0* and *TBP*	2009	[112]
Myocardial infarction	-	Mouse myocardial infarction tissue sample	*ACTB*, *B2M*, *EEF1A1*, *GAPDH*, *HPRT*, *POLR2A*, *PPIA*, *RPL13a*, *TBP* and *TPT1*	*HPRT*, *RPL13A* and *TPT1*	2011	[79]
Oral cancer	68	Saliva	*B2M*, *MT-ATP6*, *RPL30*, *RPL37A*, *RPL0*, *RPS17* and *UBC*	*MT-ATP6*, *RPL30*, *RPL37A*, *RPLP0* and *RPS17*	2016	[61]
Placenta	20	Placenta tissue	*B2M*, *GAPDH*, *HMBS*, *HPRT*, *SDHA*, *TBP*, *YWHAZ* and *LEP*	*SDHA*, *TBP* and *YWHAZ*	2004	[105]
Renal cell carcinoma	25	Tissue biopsies	*ACTB*, *ALASI*, *GAPDH*, *HMBS*, *HPRT1*, *PPIA*, *RPLP0*, *SDHA*, *TBP*, *TUBB* and *ADAM9*	*PPIA* and *TBP*	2007	[92]
Wound healing model	-	Skin and wound samples from mice	*B2M*, *TBP*, *GAPDH*, *GUSB*, *RPLP2*, *ACTB* and *18S*	*GAPDH*, *TBP* and *B2M*	2010	[107]

## Data Availability

Data are contained within the article and supplementary materials.

## Supplementary Materials

The following supporting information can be downloaded at: https://www.mdpi.com/article/10.3390/genes15020151/s1, Spreadsheet created with all the CT data of all endogenous controls and all analyzed samples in GEO database format. The file contains 4 spreadsheets: metadata template, matrix non-normalized template, matrix normalized template and fold change template.
